# Ensembl Genomes 2020—enabling non-vertebrate genomic research

**DOI:** 10.1093/nar/gkz890

**Published:** 2019-10-10

**Authors:** Kevin L Howe, Bruno Contreras-Moreira, Nishadi De Silva, Gareth Maslen, Wasiu Akanni, James Allen, Jorge Alvarez-Jarreta, Matthieu Barba, Dan M Bolser, Lahcen Cambell, Manuel Carbajo, Marc Chakiachvili, Mikkel Christensen, Carla Cummins, Alayne Cuzick, Paul Davis, Silvie Fexova, Astrid Gall, Nancy George, Laurent Gil, Parul Gupta, Kim E Hammond-Kosack, Erin Haskell, Sarah E Hunt, Pankaj Jaiswal, Sophie H Janacek, Paul J Kersey, Nick Langridge, Uma Maheswari, Thomas Maurel, Mark D McDowall, Ben Moore, Matthieu Muffato, Guy Naamati, Sushma Naithani, Andrew Olson, Irene Papatheodorou, Mateus Patricio, Michael Paulini, Helder Pedro, Emily Perry, Justin Preece, Marc Rosello, Matthew Russell, Vasily Sitnik, Daniel M Staines, Joshua Stein, Marcela K Tello-Ruiz, Stephen J Trevanion, Martin Urban, Sharon Wei, Doreen Ware, Gary Williams, Andrew D Yates, Paul Flicek

**Affiliations:** 1 European Molecular Biology Laboratory, European Bioinformatics Institute, Wellcome Genome Campus, Hinxton, Cambridge CB10 1SD, UK; 2 Department of Biointeractions and Crop Protection, Rothamsted Research, Harpenden, Hertfordshire AL5 2JQ, UK; 3 Department of Botany and Plant Pathology, Oregon State University, Corvallis, OR 97331, USA; 4 Cold Spring Harbor Laboratory, 1 Bungtown Rd, Cold Spring Harbor, NY 11724, USA; 5 USDA ARS NAA Robert W. Holley Center for Agriculture and Health, Agricultural Research Service, Ithaca, NY 14853, USA

## Abstract

Ensembl Genomes (http://www.ensemblgenomes.org) is an integrating resource for genome-scale data from non-vertebrate species, complementing the resources for vertebrate genomics developed in the context of the Ensembl project (http://www.ensembl.org). Together, the two resources provide a consistent set of interfaces to genomic data across the tree of life, including reference genome sequence, gene models, transcriptional data, genetic variation and comparative analysis. Data may be accessed via our website, online tools platform and programmatic interfaces, with updates made four times per year (in synchrony with Ensembl). Here, we provide an overview of Ensembl Genomes, with a focus on recent developments. These include the continued growth, more robust and reproducible sets of orthologues and paralogues, and enriched views of gene expression and gene function in plants. Finally, we report on our continued deeper integration with the Ensembl project, which forms a key part of our future strategy for dealing with the increasing quantity of available genome-scale data across the tree of life.

## INTRODUCTION

The goal of Ensembl Genomes is to support and facilitate non-vertebrate genome research by integrating high-quality reference genomes and annotation for every species for which this is available, providing tools and displays that allow users to interrogate and compare these genomes, and integrating with and connecting to other resources providing non-vertebrate reference genomic data. To this end, we work closely with the Ensembl project (1) to produce five additional sites, each focused on a particular non-vertebrate clade of life: bacteria (http://bacteria.ensembl.org), protists (http://protists.ensembl.org), fungi (http://fungi.ensembl.org), plants (http://plants.ensembl.org) and invertebrate metazoa (http://metazoa.ensembl.org). These sites are updated four times a year, using the same platform as, and in synchrony with, Ensembl.

Core data available for all species include genome sequence and annotations of protein-coding and non-coding genes. Transcriptional data, genetic variation and comparative analysis data are additionally available for many species. For most species, data are imported directly from the archives of the International Nucleotide Sequence Database Collaboration (INSDC) (2), the European Variation Archive (http://www.ebi.ac.uk/eva) and other open-access sources. For a few species of particular research or socioeconomic importance, additional data sets are identified and integrated. However, according to our agreement with NCBI and UCSC, we only present data on genome assemblies that have been accessioned by the INSDC.

We collaborate closely with a number of other international resources in various domains of life, including Gramene (http://www.gramene.org) (3) for plants, VectorBase (http://www.vectorbase.org) (4) for invertebrate vectors of human pathogens and WormBase (http://www.wormbase.org) (5) for nematodes and flatworms. Together, we develop common data sets and integration/analysis workflows, which are made available through both Ensembl Genomes and project-specific websites and services.

## ACCESS

All data in the resource are open access and can be explored in multiple ways. Interactive and visual access is provided through our websites. A key component of these sites is our embedded genome browser, enabling users to scroll through a graphical representation of a DNA molecule at different levels of resolution and visualize various types of annotations as tracks on the browser, including gene models, genomic variants, repetitive elements and homology sequences aligned to the genome. We also display functional information on genes and gene products, imported from the UniProt Knowledgebase (6) and analysis of peptide sequence (using the classification tool InterProScan (7)). Various tools for text and sequence search, data upload and data analysis are provided, allowing users to examine their own data in the context of the reference sequence and annotation.

All data are made available as database dumps, and common data sets (e.g. DNA, RNA and protein sequence sets, and sequence alignments) can be directly downloaded in bulk via FTP (ftp://ftp.ensemblgenomes.org) in a variety of formats. For certain data types, the web application utilizes data files stored in archival resources (such as the European Nucleotide Archive), avoiding the need for database builds and improving the speed of response. These files are also made available to users.

Data are also made available through a series of data warehouses, optimized around common (gene- and variant-centric) queries, using the BioMart data warehousing system (8). The BioMart framework provides a series of interfaces, including web-based query building tools, accessible at each of the Ensembl Genomes eukaryotic portals, and a variety of other interfaces for interactive and programmatic access. BioMarts are not currently available for Ensembl Bacteria.

In recognition of the fact that certain use cases require access to a single version of the data and browser for longer that the ∼3-month life cycle of an Ensembl release, we designate certain releases as ‘archive’ releases and provide continued access to the website beyond when they have been superseded by new releases. Archive releases to date are 37 (e.g. http://eg37-plants.ensembl.org) and 40 (e.g. http://eg40-fungi.ensembl.org).

Finally, for programmatic access, we provide a comprehensive language-agnostic RESTful Application Programming Interface (API). A significant update this year has been the consolidation of the APIs for Ensembl and Ensembl Genomes into a single service (https://rest.ensembl.org), for the first time providing access to both vertebrate and non-vertebrate data through a single programmatic interface. A training manual for the consolidated API can be found at https://www.ebi.ac.uk/training/online/course/ensembl-rest-api.

## NEW AND IMPROVED GENOMES

The past 2 years have seen continued growth in our genome collections (see Table 1). Contents of Ensembl Bacteria have been frozen while our focus has been on other divisions in this period. We are working with partners to develop a coherent strategy for prokaryotes, especially in the context of the growing availability of metagenomic data, and anticipate significant progress on this in the next year.

**Table 1.** Ensembl Genomes growth 2017–19

		Number of genomes				
Release	Date	Bacteria	Protists	Fungi	Plants	Metazoa
37	September 2017	44 048	189	811	45	68
45	September 2019	44 048	237	1014	67	78
Increase		0	48	203	22	10

Of the 250 genomes added in Ensembl Fungi and Ensembl Protists, significant additions include *Puccinia coronata*, a new and fast-spreading pathogen of barley and oats; *Phytophthora megakarya*, an oomycete pathogen causing black pod disease in cocoa trees; and the ice-loving diatom *Fragilariopsis cylindrus*, found in Arctic and Antarctic seawater.

In metazoa, notable additions have been *Folsomia candida* and *Orchesella cincta* as examples of organisms important for the assessment of soil quality; *Bombus terrestris* (the buff-tailed bumblebee), a declining insect species; *Daphnia magna*, which is used in studying toxicity (such as polystyrene nanoplastics, lead and pesticides) in aquatic environments; *Teleopsis dalmanni* (the stalk eyed fly), a model organism for studying sexual selection mechanisms; and the disease vectors *Leptotrombidium deliense* (scrub typhus) and *Culicoides sonorensis* (bluetongue arbovirus in livestock).

In plants, we have significantly increased coverage of asterids, including *Actinidia chinensis* (kiwifruit), *Coffea canephora* (robusta coffee), *Cynara cardunculus* (globe artichoke), *Daucus carota* (carrot), *Capsicum annuum* (hot pepper) and *Helianthus annuus* (sunflower). Elsewhere in the taxonomy, we have included *Arabidopsis halleri*, a brassica model for ecological genomics due to its heavy metal hyperaccumulation ability; and *Marchantia polymorpha*, a liverwort from a basal land plant lineage with a haploid genome that exhibits low genetic redundancy compared to other land plants.

As well as adding new species, we regularly update selected genome assemblies as they are released by collaborators or INSDC archives. A notable example is the new version of the hexaploid bread wheat genome (*Triticum aestivum*) from the International Wheat Genome Sequencing Consortium (RefSeq v1.0) (9). This has been accompanied by updates to the related diploid progenitor species *Aegilops tauschii* and the tetraploid *Triticum dicoccoides* (emmer wheat), alongside the addition of tetraploid *Triticum turgidum* (durum wheat). The previous wheat assembly, version TGACv1, can still be queried in the archived Ensembl release http://eg37-plants.ensembl.org.

## PRIMARY ANNOTATION

Ensembl Genomes primary annotation (i.e. protein-coding and non-coding gene structures) derives from four main sources: (i) expert manual annotation, as part of specific funded collaborations; (ii) computational annotation pipelines, such as MAKER (10) and the Ensembl gene annotation system (11), deployed locally; (iii) annotation sets provided by collaborators and submitters (usually as GFF3 files); and (iv) annotation deposited directly on the INSDC records by the original genome project. We maintain a page for each genome that documents the provenance of the primary annotation, as well as other data sets we have integrated (e.g. http://metazoa.ensembl.org/Anopheles_gambiae/Info/Annotation).

Through our involvement in the WormBase project, we provide expert curation of the reference gene set for *Caenorhabditis elegans* and a number of other well-studied nematodes. We have recently added *Trichuris muris* (mouse model for trichuriasis) to the set of genomes we curate. We also facilitate manual curation by community experts via our participation in VectorBase by deploying an instance of the online annotation editing tool Apollo (12), and have developed a robust workflow for periodically folding community-provided annotations back into the canonical gene set. We have extended this approach to selected fungal pathogen genomes by facilitating collaborations between geographically dispersed research teams. We enable these communities to redefine the gene set for these pathogens by providing an instance of Apollo and training in manual annotation best practice. For example, we worked with 42 members of the *Botrytis cinerea* research community from 10 institutions in 8 countries. This group curated the entire gene set in under 7 months, making edits to 11 612 gene structures and adding 35 new genes. A similar project for *Blumeria graminis* f. sp. *hordei* involved 4 members of the *B. graminis* community from 2 institutions, and we are currently engaged with 26 experts from 8 institutions in revising annotation for the wheat pathogen *Zymoseptoria tritici*. This latter effort has greatly helped disambiguate neighbouring genes in *Z. tritici* that had been wrongly merged in the automatic predictions. We are continuously improving our training material around gene curation using Apollo using feedback from our community contributors, which will be used to support subsequent projects.

For the majority of genomes we host (100% of bacteria and 85% of eukaryotic genomes), primary annotation has been imported directly from the INSDC records using our GenomeLoader pipeline. GenomeLoader queries the ENA directly and imports assemblies and corresponding annotation into Ensembl MySQL databases for a given clade. In the past year, we have re-engineered the pipeline and now use Ensembl workflow manager eHive (13) to orchestrate and distribute the loading of many genomes in parallel across a multiprocessor cluster, improving scalability.

## ANNOTATION OF GENE EXPRESSION

**Figure 1.**
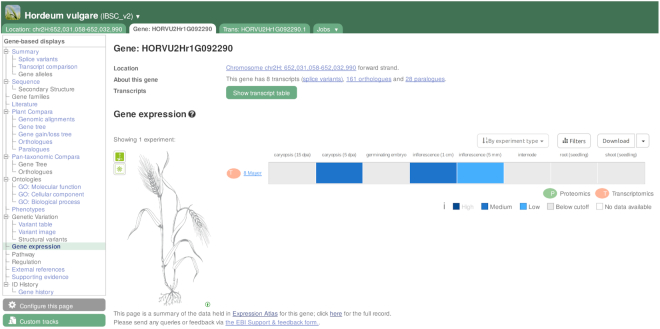
Baseline expression of barley gene HORVU2Hr1G092290, available as ‘Gene expression’ on the left menu. This feature is currently supported for the following plant species: *Arabidopsis thaliana*, *Brassica rapa*, *Brachypodium distachyon*, *Hordeum vulgare*, *Glycine max*, *Oryza sativa*, *Populus trichocarpa*, *Setaria italica*, *Solanum tuberosum*, *Sorghum bicolor*, *Triticum aestivum* and *Zea mays*.

We previously reported on a pipeline we had begun work on in collaboration with the EMBL-EBI Gene Expression Atlas (GxA) (14) for the automatic analysis and display of RNA-Seq gene expression data. Briefly, the pipeline (i) identifies appropriate RNA-Seq assays in the INSDC; (ii) aligns them to the corresponding reference genome to produce CRAM (15) files; (iii) submits the CRAM files back to ENA for persistent archival; and (iv) registers new studies and the experiments they comprise in the Track Hub Registry (http://trackhubregistry.org), facilitating ease of discoverability and visualization using the Ensembl Genomes browsers. As a further strengthening of the collaboration with the Gene Expression Atlas and Gramene, we now curate metadata for high-value expression studies in key plant species. Gene expression according to these curated studies can be explored via the Gene Expression widget in our gene report pages, featuring interactive anatograms for 12 species, including *Arabidopsis thaliana*, *Brachypodium distachyon* or wheat (see example in Figure 1). Curation involves mapping user-submitted sample descriptions to a standardized list of terms from the Experimental Factor Ontology (16), facilitating integration and comparison across different studies. A fuller exploration of the data is available from the GxA site (http://www.ebi.ac.uk/gxa).

## GENOME VARIATION AND GENE FUNCTION ANNOTATION

**Figure 2.**
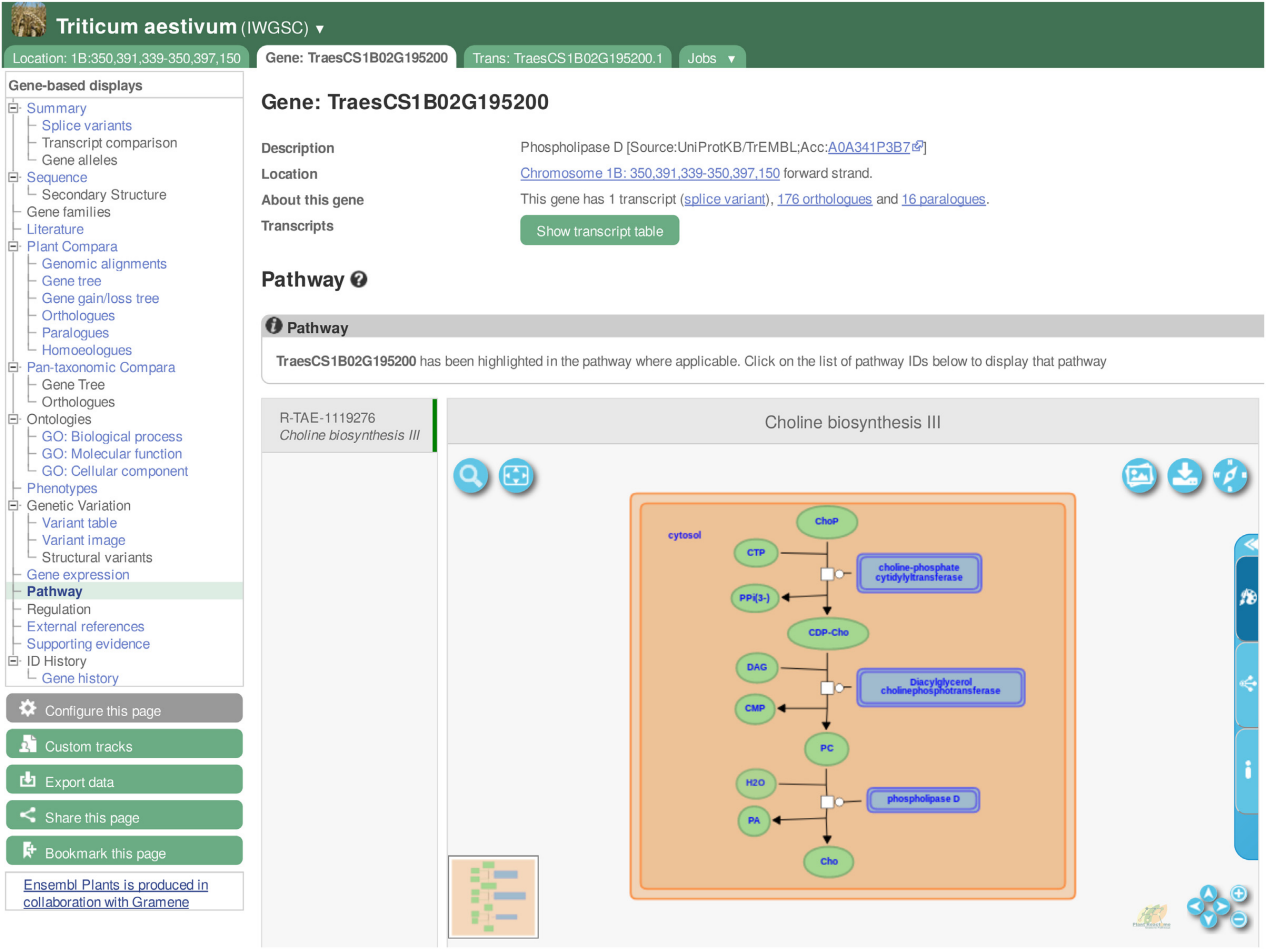
Wheat gene TraesCS1B02G195200 (phospholipase D) is annotated as an enzyme involved in choline biosynthesis. This diagram is available as ‘Pathway’ on the left menu. This is an orthology-based projection from rice gene OsPLDalpha1, which was curated from the literature at the Plant Reactome database.

A fundamental feature of Ensembl Genomes is the integration of diverse data sets to enhance our understanding of gene function. For plants, we have collaborated with Gramene to expand our annotation of genes to metabolic pathways in 54 species. This is done by projecting cross-references to reactions in the Plant Reactome database (17) via orthology, capitalizing on the curation of 50 new rice pathways in the past 2 years. The pathways and reactions can be explored via an embedded widget on gene report pages (see Figure 2). Furthermore, as part of an Elixir (https://elixir-europe.org) implementation study on microbial systems biology, we have annotated 22 million bacterial genes with cross-references to the Rhea database (18) of biochemical reactions (https://www.rhea-db.org).

We also have further improved our integration with PHI-base (19), a manually curated database of host–pathogen interactions (http://www.phi-base.org), refining the way in which we extrapolate annotations to closely related microbial species. Our new pipeline has enabled us to increase the number of genomes with PHI-base annotations by a factor of 10, and the number of genes by a factor of 7. We now have PHI-base annotations to over 14 000 genes in over 1000 microbial genomes using both direct matches to records in PHI-base and sequence similarity.

We also integrate and annotate genetic variants for a small number of well-studied species. A particular recent highlight in this area has been our integration of over 16 million variants in bread wheat. Much of these data have come from genotyping arrays and sequenced EMS-induced mutations in cultivars, imported from CerealsDB (20), but we also represent the differences between the A, B and D component genomes as interhomoeologous variants. We have annotated the effect of these variants using the Ensembl Variant Effect Predictor (21) and added information on the potential damage of missense protein-coding mutations using SIFT (22).

## COMPARATIVE GENOMICS

We continue to provide a rich suite of tools for comparing species, at the level of both the whole genome and individual genes. For genome-level comparisons, we provide numerous pairwise whole-genome alignments, deploying a number of different methods depending on the clade and divergence. A notable addition in the past 2 years has been a Cactus (23) multiple sequence alignment for our collection of nematode genomes. Among plants, we now have pairwise alignments among the A, B and D subgenomes of hexaploid wheat, which support the comparison of homoeologues in their genomic context.

For gene-level comparisons, we classify genes into families, compute an evolutionary tree for each family and use the tree to infer orthology and paralogy relationships for pairs of genes. We use the Ensembl gene-tree pipeline (24) to perform this task, individually parameterized for each division. The pipeline has been extended to be polyploidy-aware, and predicts homoeologues in the hexaploid bread wheat and tetraploid emmer and durum wheat by considering each component genome as if it was from an independent species.

A distinguishing feature of Ensembl Genomes is our pan-division gene trees, where we have selected just under 200 representative genomes from across the tree of life (including vertebrates) to calculate gene trees and orthologues/paralogues. The set is manually curated, adding new genomes when they occupy a part of the taxonomy that is not already represented. Currently, around 60% of the selected genomes are bacteria, reflecting greater phylogenetic spread in prokaryotes. A representative example of a pan-taxonomic gene tree for a universally conserved ribosomal protein can be found at http://plants.ensembl.org/Multi/GeneTree/Image/pan_compara?gt=EGGT00960000274575.

Across the Ensembl collection of resources, we therefore provide a number of different sets of gene trees. Because the sets of genes in each analysis are not completely disjoint, it has been the case in the past that, for example, a *C. elegans* gene could be clustered into different families in the vertebrate, non-vertebrate metazoa and pan-division analysis, due to the stochastic nature of the clustering process. To mitigate for this, we have recently migrated to using a new version of the Ensembl gene-tree pipeline that uses a common set of hidden Markov model libraries from PANTHER (25) and TreeFam (26) for initial classification. This has increased the consistency of classification between per-division analyses and also between different releases for each division. An important advantage of the new method is that it allows more fine-grained classification of genes via the use of PANTHER subfamilies. Trees for each subfamily are linked together into family-level ‘supertrees’, which allows us to define orthology and paralogy relationships at both subfamily and family levels.

## FUTURE PLANS

While automated import of genomes and annotation from INSDC and other public repositories will continue at scale and account for the majority of the volume of data we serve, we are also committed to the model of deeper curation and integration of specific data sets and types as part of domain-specific collaborations. For example, in collaboration with PHI-base (19), we have recently started a project to deepen our curation and integration of pathogen–host interactions of medical and agricultural importance. A component of this work involves the development of a curation system to represent and capture these interactions at the molecular level.

For resources that integrate genomic data, it is becoming increasingly necessary to provide scalable and meaningful mechanisms for comparing genomes of closely related species and strains. This was demonstrated by our Ensembl-4-Breeders workshop, in which we gathered together a group of plant breeders and geneticists to gather information on their genomics use cases. Clear feedback from the breeding community was that a single genome (and associated gene set) is often insufficient for crops, and that the representation of multiple individuals or strains would be of great value. The 10+ wheat genome project (http://www.10wheatgenomes.com) aims to generate dozen wheat cultivars to capture the variation in genome structure and gene complement within the species. We will integrate these data as they become available, providing tools to compare the cultivars via whole-genome alignments and gene trees. We will also track the state of the art of ‘pan-genome’ data representations (27) and associated tools for alignment and annotation, and adopt standards as they emerge.

The rate of growth of genomic data in the public domain continues to accelerate. Ambitious large-scale sequencing projects like the Darwin Tree of Life and the Earth BioGenome Project (28) will place even greater demands on both Ensembl and Ensembl Genomes as we seek to provide broad coverage and detailed analysis of the genomic space. Moreover, as genomic data are increasingly available for groups of taxonomically distant interacting species and even for entire ecosystems, the value of uniform analysis presentation across the taxonomic space will increase. Common workflows, efficient pipelines and well-thought-out visualization strategies will be necessary.

With this in mind, the public REST APIs of Ensembl and Ensembl Genomes have already been merged, and internal standardization of data processing pipelines is being pursued wherever possible. We are planning to bring the two projects together in a single website (to be rolled out throughout 2020 in a phased manner). This will be supplemented by specialized subportals emphasizing certain data (for instance, data related to hosts, vectors and pathogens) for particular user communities, emphasizing interactions between species in their natural ecosystems and in disease processes. We believe this will pave the way for a new type of data exploration that will bring closer organisms from different divisions, such as bacterial symbionts, mycorrhizal fungi and their hosts. This integration will have an impact on applications such as drug target discovery and breeding.
